# Using Ticagrelor to Prevent Recurrent Type 1 and Type 2 Myocardial Infarctions: Boon or Bane?

**DOI:** 10.1161/JAHA.118.010996

**Published:** 2018-11-20

**Authors:** John A. Bittl

**Affiliations:** ^1^ Interventional Cardiology Group Florida Hospital Ocala Ocala FL

**Keywords:** Editorials, demand myocardial infarction, dual antiplatelet therapy, myocardial oxygen demand, P2Y12, percutaneous coronary intervention

## Abstract

See Editorial by https://doi.org/10.1161/JAHA.118.009260

## Introduction

There are ≈580 000 new acute myocardial infarctions (MI) and 210 000 recurrent MIs in the United States every year.^1^ Because platelets play a central role in the pathogenesis of MI,^2^ it is possible that adding a potent oral antiplatelet agent like ticagrelor to low‐dose aspirin prevents more MIs than using aspirin alone.

To determine how effectively ticagrelor prevents recurrent MIs, Bonaca and colleagues analyzed data from the PEGASUS‐TIMI 54 (Prevention of Cardiovascular Events in Patients With Prior Heart Attack Using Ticagrelor Compared With Placebo on a Background of Aspirin–Thrombolysis in Myocardial Infarction 54) trial.^3^ The original trial enrolled >21 000 patients with a history of MI in the past 1 to 3 years and randomized them in a 1:1:1 manner to low‐dose aspirin or aspirin plus 2 different doses of ticagrelor (90 and 60 mg BID). In the original publication, the investigators presented a landmark trial that described the ability of ticagrelor to reduce composite cardiovascular events in stable patients with a history of previous MI.^3^ In the current issue of the *Journal of the American Heart Association* (*JAHA*),^4^ the investigators focus on the ability of ticagrelor to reduce the type and size of MIs and make several new and important observations about secondary prevention.

The investigators found that a total of 1042 MIs occurred in 898 of the 21 162 randomized patients over a median follow‐up of 33 months.^4^ This translated into a rate of recurrent MI of 1.7% per year. The investigators found that 792 of the 1042 MIs (76%) were spontaneous (type 1), and 224 (21%) were caused by ST‐segment–elevation MI. The investigators found that 138 MIs (13%) were caused by an imbalance in myocardial oxygen supply and demand (type 2), and 98 (9%) were caused by stent thrombosis (type 4b). Events associated with sudden death (type 3), percutaneous coronary interventions (type 4a), or coronary artery bypass graft procedures (type 5) each accounted for <1% of the MIs.

The study found that ticagrelor reduced all MIs over the entire study period by 17% (4.47% versus 5.25%; hazard ratio [HR], 0.83; 95% confidence interval [CI], 0.72–0.95; *P*=0.0055). The major benefit of ticagrelor was confined to type 1 MIs, with the most significant effect being a 40% reduction in ST‐segment–elevation MIs (HR, 0.60; 95% CI, 0.46–0.78; *P*=0.0002) and a 31% reduction in MIs with a peak troponin of ≥100 times the upper limit of normal (HR, 0.69; 95% CI, 0.53–0.92; *P*=0.0096).^4^ It is plausible that ticagrelor prevents recurrent type 1 MIs because platelets are activated by atherosclerotic plaque disruption (rupture or erosion) in this condition,^5^ and patients with a history of MI have a sustained heightened risk for recurrent atherothrombotic events.^2^

The investigators suggested that ticagrelor could also reduce type 2 MIs,^4^ but there are several reasons to question this conclusion. First, the reduction was not statistically significant (HR, 0.82; 95% CI, 0.57–1.18; *P*=0.28). Although an argument could be made that the nonsignificant findings were caused by a type 2 statistical error, a bayesian approach would have produced the same conclusion. In the absence of a plausible mechanism, the bayesian approach would combine a skeptical prior with the borderline evidence and generate a posterior to show that the benefit of ticagrelor is improbable.^6^ From pathogenetic principles, it does not seem plausible that ticagrelor could prevent a condition like sepsis or bleeding that leads to an imbalance in myocardial oxygen supply and demand. Moreover, potent antiplatelet therapies like ticagrelor are contraindicated or ineffective in many of the conditions associated with demand MIs, such as severe anemia, noncardiogenic shock, or respiratory failure.

This is not a criticism of the present analysis, but the concept of type 2 MIs has engendered much confusion since the publication of the original universal definition.^5^ When a coauthor of the present report^4^ writes in an authoritative textbook that,^7^ “Estimates of the proportion of MIs that are type 2 vary widely from 3.5% to 72%, depending on the setting and approaches to diagnostic categorization,” he identifies a challenge in diagnostic coding and a crisis in cardiac care.

The introduction of high‐sensitivity troponin assays has doubled the incidence of type 2 MIs,^8^ which partly reflects a common practice of equating troponin elevation with the diagnosis of MI. Although myocardial injury *or* necrosis can lead to troponin elevation, the diagnosis of MI requires the presence of myocardial necrosis. A strict distinction between injury and necrosis is important because injury may be reversible, whereas necrosis is not. The conflation of injury and necrosis is a solecism, which arises from a breach in understanding of pathophysiological principles, leading to much confusion. The primacy of using troponin elevation for the diagnosis of MI in a heterogeneous group of conditions is analogous to stating that fever is pathognomonic for infection. Type 2 MIs are caused by too many different conditions to be clinically meaningful when lumped together. It will be important for future iterations of the universal definition to discuss whether type 2 MI is a useful clinical category that defines a coherent syndrome caused by a single pathogenetic mechanism, amenable to a uniform treatment, associated with a well‐defined prognosis or preventable by using a single therapeutic class of medications, such as platelet P2Y12 inhibitors.

Compared wtih type 2 MIs, type 4b MIs (stent thrombosis) have a more comprehensible pathogenesis, but the authors of the present analysis found that type 4b MIs (stent thrombosis) were not significantly reduced by ticagrelor (HR, 0.78; 95% CI, 0.52–1.18; *P*=0.25), and events associated with sudden death (type 3), percutaneous coronary intervention (type 4a), or coronary artery bypass graft procedures (type 5) occurred with an incidence that was too low to draw conclusions.^4^ Although ticagrelor produced an apparent ≈17% reduction in MI across the entire spectrum, only the reduction in type 1 MIs met the accepted definition of statistical significance, which is a reminder that translating statistical findings into everyday English can be difficult.^9^

Given that the benefit of ticagrelor was restricted to reducing type 1 MIs, clinicians may ask: How effective was ticagrelor? Confining the analysis to relative measures, such as HRs, tends to produce a distorted sense of proportion and makes it difficult to translate the results into clinical practice. Using absolute event rates gives clinicians a better sense of what works in cardiovascular medicine.^10^ If it is assumed that the treatment effect is consistent from one year to the next, the absolute differences in annual event rates can be used to calculate the numbers needed to treat.^11^ Comparing numbers needed to treat for 2° prevention (Table) reveals that using ticagrelor is on par with using aspirin for secondary prevention,^12^ high‐intensity compared with low‐intensity statins to prevent nonfatal MI,^13^ or the proprotein convertase subtilisin/kexin type 9 (PCSK9) inhibitor arilocumab to prevent recurrent MI.^14^ Because the benefit of ticagrelor was confined to type 1 MIs, it can be shown that 461 (95% CI, 280–2617) must be treated with ticagrelor to prevent a single type 1 MI, and ≈700 patients (95% CI, 516–1267 patients) must be treated each year to prevent a single ST‐segment–elevation MI.

**Table 1:** NNTs per Year to Prevent Recurrent MI

Treatment	Control Rate, %	NNT (95% Confidence Interval)	Reference
Per annum			
Ticagrelor	1.7	351 (239–747)	4
Aspirin	1.2	278 (216–432)	12
Intensive statin	1.5	290 (248–351)	13
After 2.8 y			
Arilocumab	7.6	101 (61–355)	14

MI indicates myocardial infarction; NNT, number needed to treat.

Analyzing the benefit of ticagrelor tells only part of the story. Evaluating the adverse effects and bleeding caused by ticagrelor was beyond the scope of the present report,^4^ but knowledge of adverse effects is important to translate results into clinical practice. In the original trial,^3^ dyspnea led to study drug withdrawal 5 to 9 times more often with ticagrelor than with placebo. Life‐threatening bleeding occurred 2 to 3 times more often with ticagrelor than with aspirin alone. The number needed to harm each year with the 90‐mg dose to cause a TIMI major bleed was 169 (95% CI, 106–296), and the number needed to harm with the 60‐mg dose was 216 (95% CI, 129–417). When the number needed to harm is less than the number needed to treat, harm may be more likely than benefit.

Despite the quibbles, Bonaca and colleagues are commended for performing a novel and insightful analysis describing the ability of ticagrelor to reduce type 1 MIs, ST‐segment–elevation MIs, and large MIs.^4^ Because of its ability to reduce spontaneous MIs caused by atherothrombotic mechanisms, ticagrelor may have a net benefit beyond the recommended 1 year of therapy in patients with previous MI who have low bleeding risk and above‐average ischemic risk, as defined by the PEGASUS‐TIMI 54 trial eligibility criteria. In an effort to find a better balance between ischemic benefit and bleeding risk in the broader population of patients with a history of MI, further investigation is needed to compare potent antiplatelet agents as monotherapy with aspirin alone for the prevention of ischemic events and bleeding in patients who undergo percutaneous coronary intervention or have a history of MI.^15^

## Disclosures

None.

## References

[1] Benjamin EJ , Blaha MJ , Chiuve SE , Das SR , Deo R , de Ferranti SD , Floyd J , Fornage M , Gillespie C , Isasi CR , Jiménez MC , Jordan LC , Judd SE , Lichtman JH , Lisabeth L , Liu S , Longenecker CT , Mackey RH , Matsushita K , Mozaffarian D , Mussolino ME , Nasir K , Neumar RW , Palaniappan L , Panday DK , Thiagarajan RR , Reeves MJ , Ritchey M , Rodriguez CJ , Roth GA , Rosamond WD , Sasson C , Towfighi A , Tsao CW , Turner MB , Virani SS , Voeks JH , Willey JZ , Wilkins JT , Wu JH‐Y , Alger HM , Wong SS , Muntner P ; on behalf of the American Heart Association Statistics Committee and Stroke Statistics Subcommittee . Heart disease and stroke statistics—2017 update: a report from the American Heart Association. Circulation. 2017;135:e146–e603. 28122885 10.1161/CIR.0000000000000485 PMC5408160

[2] Bonaca MP , Sabatine MS , Morrow DA . Antiplatelet therapy after myocardial infarction In: Morrow DA, ed. Myocardial Infarction: A Companion to Braunwald's Heart Disease. St Louis, MO: Elsevier; 2017:434–448.

[3] Bonaca MP , Bhatt DL , Cohen M , Steg PG , Storey RF , Jensen EC , Magnani G , Bansilal S , Fish MP , Im K , Bengtsson O , Ophuis TO , Budaj A , Theroux P , Ruda M , Hamm C , Goto S , Spinar J , Nicolau JC , Kiss RG , Murphy SA , Wiviott SD , Held P , Braunwald E , Sabatine MS ; for the PEGASUS‐TIMI 54 Steering Committee and Investigators . Long‐term use of ticagrelor in patients with prior myocardial infarction. N Engl J Med. 2015;372:1791–1800. 25773268 10.1056/NEJMoa1500857

[4] Bonaca MP , Wiviott SD , Morrow DA , Steg PG , Hamm C , Bhatt D , Storey R , Cohen M , Kuder J , Im KA , Magnani G , Budaj A , Theroux P , Nicolau J , Parkhomenko A , Lopez‐Sendon JL , Dellborg M , Diaz F , Goudev A , Jensen E , Johanson P , Braunwald E , Sabatine MS . Reduction in subtypes and sizes of myocardial infarction with ticregrelor in PEGASUS‐TIMI 54. J Am Heart Assoc. 2018;7:e009260 DOI: 10.1161/JAHA.118.009260. 30571502 PMC6404436

[5] Thygesen K , Alpert JS , Jaffe AS , Chaitman BR , Bax JJ , Morrow DA , White HD , Mickley H , Crea F , Van de Werf F , Bucciarelli‐Ducci C , Katus HA , Pinto FJ , Antman EM , Hamm CW , De Caterina R , Januzzi JL Jr , Apple FS , Garcia MAA , Underwood SR , Canty JM Jr , Lyon AR , Devereaux PJ , Zamorano JL , Lindahl B , Weintraub WS . Fourth universal definition of myocardial infarction (2018). J Am Coll Cardiol. 2018;72:2231–2264. 30153967 10.1016/j.jacc.2018.08.1038

[6] Bittl JA , He Y . Bayesian analysis: a practical approach to interpret clinical trials and create clinical practice guidelines. Circ Cardiovasc Qual Outcomes. 2017;10:1–11. 10.1161/CIRCOUTCOMES.117.003563 PMC6421843 28798016

[7] Morrow DA , Braunwald E . Classification and diagnosis of acute coronary syndromes In: Morrow DA, ed. Myocardial Infarction: A Companion to Braunwald's Heart Disease. St Louis, MO: Elsevier; 2017:1–10.

[8] Roffi M , Patrono C , Collet J‐P , Mueller C , Valgimigli M , Andreotti F , Bax JJ , Borger MA , Brotons C , Chew DP , Gencer B , Hasenfuss G , Kjeldsen K , Lancelotti P , Landmesser U , Mehilli J , Mukherjee D , Storey R , Windecker S . 2015 ESC guidelines for the management of acute coronary syndromes in patients presenting without persistent ST‐segment elevation. Eur Heart J. 2015;37:267–315.26320110

[9] Pocock SJ , Ware JH . Translating statistical findings into plain English. Lancet. 2009;373:1926–1928. 19375158 10.1016/S0140-6736(09)60499-2

[10] Bittl JA , Maron DJ . Using absolute event rates to see what works in cardiovascular medicine. J Am Coll Cardiol. 2017;70:1376–1378. 28882236 10.1016/j.jacc.2017.07.764

[11] Cates CJ . Simpson's paradox and calculation of number needed to treat from meta‐analysis. BMC Med Res Methodol. 2002;2:1. 11860604 10.1186/1471-2288-2-1 PMC65632

[12] Antithrombotic Trialists’ Collaboration . Aspirin in the primary and secondary prevention of vascular disease: collaborative meta‐analysis of individual participant data from randomised trials. Lancet. 2009;373:1849–1860. 19482214 10.1016/S0140-6736(09)60503-1 PMC2715005

[13] Cholesterol Treatment Trialists’ (CTT) Collaboration . Efficacy and safety of more intensive lowering of LDL cholesterol: a meta‐analysis of data from 170 000 participants in 26 randomised trials. Lancet. 2010;376:1670–1681. 21067804 10.1016/S0140-6736(10)61350-5 PMC2988224

[14] Pocock SJ , Collier TJ . Critical appraisal of the 2018 ACC scientific sessions late‐breaking trials from a statistician's perspective. J Am Coll Cardiol. 2018;71:2957–2969. 29753563 10.1016/j.jacc.2018.04.015

[15] Gargiulo G , Windecker S , Vranckx P , Gibson CM , Mehran R , Valgimigli M . A critical appraisal of aspirin in secondary prevention: is less more? Circulation. 2016;134:1881–1906. 27920074 10.1161/CIRCULATIONAHA.116.023952

